# Relative weighting of acoustic information during mating decisions in grasshoppers indicates signatures of sexual selection

**DOI:** 10.1007/s00359-017-1200-x

**Published:** 2017-07-21

**Authors:** Jan Clemens, Jennifer Aufderheide, Bernhard Ronacher

**Affiliations:** 1 Princeton Neuroscience Institute, Princeton University, Washington Road, Princeton, NJ 08540 USA; 2 Behavioral Physiology Group, Department of Biology, Humboldt Universität zu Berlin, 10115 Berlin, Germany

**Keywords:** Acoustic communication, Decision-making, Sexual selection, Mate choice, Computational modelling

## Abstract

The decision with whom to mate is crucial in determining an individual’s fitness and is often based on the evaluation of visual or acoustic displays produced during courtship. Accordingly, the algorithms for evaluating such courtship signals are shaped by sexual selection and should reflect the expected benefits and costs of mating: signals bearing heterospecific features should be rapidly rejected, since mating would produce no fertile offspring, while signals resembling conspecific ones should be weighted proportional to mate quality. We test these hypotheses in females of the grasshopper *Chorthippus biguttulus* who assess males by their song, which is produced as a sequence of subunits with species and individual specific signatures. We present mixed sequences of subunits with conflicting cues and use a computational model of decision-making to infer how sensory information is weighted and integrated over the song. Consistent with our hypothesis, females do weight sensory cues according to the expected fitness benefits/costs: heterospecific subunits are weighted particularly negatively and lead to a rejection of the male early in the song. Conspecific subunits are weighted moderately, permitting a more complete evaluation of the full song. However, there exists an overall negative bias against mating, possible causes of which are discussed.

**Electronic supplementary material:**

The online version of this article (doi:10.1007/s00359-017-1200-x) contains supplementary material, which is available to authorized users.

## Introduction

The choice of a mating partner belongs to the most crucial decisions in an animal’s life. In line with their high investment into large gametes, females, as a rule, are particularly choosy and tend to select high-quality mates (Andersson 1994; Andersson and Simmons 2006). Females base their mate choice on sensory information—often in the form of elaborate visual or acoustic displays produced by the males during courtship (Gerhardt and Huber 2002). The rules by which females evaluate the sensory information from these displays are likely the outcome of sexual selection on choice behavior. That is, the value accrued to a courtship signal should reflect the expected benefits and costs of mating with the sender (Gerhardt and Huber 2002; Qvarnström and Price 2001; Kokko et al. 2002; Head et al. 2005). Signals that are indicative of a different species should be rejected, since mating with heterospecifics does normally not yield fertile offspring (Naisbit et al. 2002; Safi et al. 2006; Ritchie 2007; McDermott and Noor 2010). On the other hand, signals usually produced by conspecifics should be evaluated according to their similarity with the “ideal” ones to favor selection of the most high-quality males (Hoikkala et al. 1998; Qvarnström and Price 2001; Gerhardt and Huber 2002; Neff and Pitcher 2005). Moreover, the dynamics of the decision process should be tuned to support efficient decision-making—driving fast decisions if the signal unequivocally marks the sender as inappropriate but evaluating sensory information in more detail if information is ambiguous (Tajima et al. 2016).

Females of the grasshopper *Chorthippus biguttulus* use the songs of males as an indicator of species, gender, and quality (von Helversen 1972; Kriegbaum 1989; von Helversen and von Helversen 1997; Klappert and Reinhold 2003; Safi et al. 2006; Stange and Ronacher 2012). In this bidirectional acoustic communication system, females respond to a male’s calling song with a response song if they are inclined to mate and if the male’s song exhibits species-specific and attractive features (von Helversen and von Helversen 1997; Balakrishnan et al. 2001; Ronacher and Stange 2013). In an earlier investigation, we took advantage of this bidirectional communication and confronted grasshopper females with equivocal quality information, using models of male songs that consisted of pseudorandom sequences of attractive and unattractive subunits including those of heterospecifics (Clemens et al. 2014). The females’ responses were analyzed with a drift diffusion model, in which positive and negative sensory information is accumulated in a noisy manner. By this approach, we could directly infer: (1) the values attributed to attractive and unattractive subunits as given by their weights in the model, and (2) the dynamics of the decision to respond to a male song. Surprisingly, sensory evidence provided by the attractive subunits was effectively neutral in the model and thus not able to drive a positive decision—a female response song. By contrast, negative subunits dominated the decision process to the effect that a few negative subunits in the beginning of a song effectively vetoed the behavioral response. We interpreted these results as follows: the negative subunits did unambiguously indicate an unfit male and hence rapidly suppressed the females’ response. This is in line with recent evidence in this grasshopper species that mating with a heterospecific partner would have fatal consequences for a female’s fitness, and, therefore, must be avoided (Gottsberger and Mayer 2007; Finck and Ronacher 2017).

However, if we assume that the rules of decision-making during song evaluation in grasshoppers are shaped by sexual selection, we expect a more nuanced assessment of song information. Heterospecific subunits should have a particularly strong negative weight to avoid a waste of mating resources (Neff and Pitcher 2005; Finck and Ronacher 2017). On the other hand, subunits only moderately deviating from the species-typical one should be weighted in a graded manner according to their dissimilarity from an “ideal” song. There should also exist clearly attractive subunits, not just the rather neutral one found previously (Clemens et al. 2014), leading to positive decisions being more strongly driven by sensory information if a male produces particularly attractive subunits (see Balakrishnan et al. 2001).

**Fig. 1:**
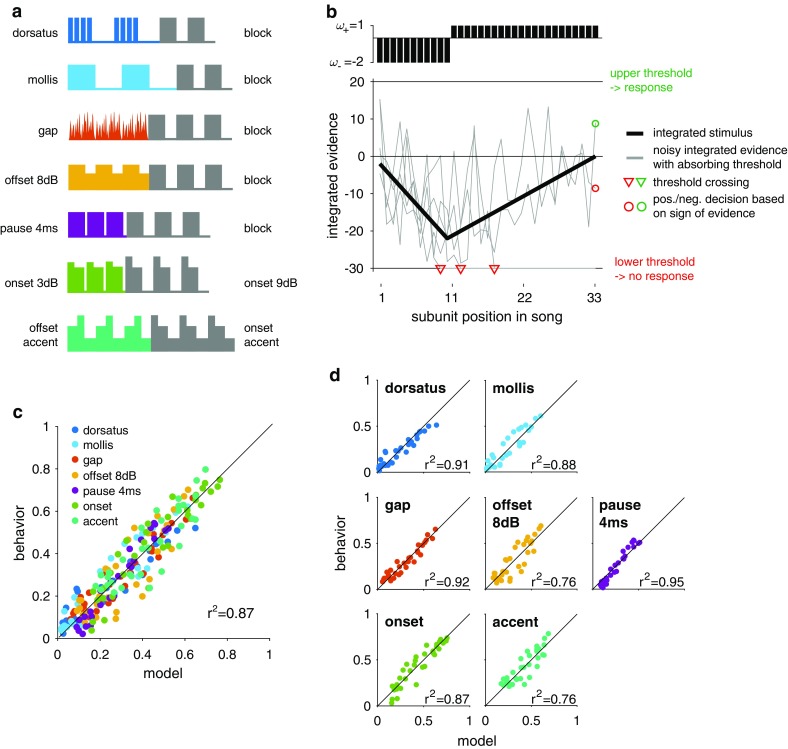
Stimuli used and drift diffusion model. **a** Pattern of the subunits used in the behavioral experiments. Subunits were always tested in pairs—the two subunits in a pair were arranged in 32 different sequences of 33 subunits, such that the relative amount of each subunit in different parts of the sequence was diverse (see “Methods” and Table 1 for details). **b** Schematic of the drift diffusion model. Each subunit (*black bars*) in the song is assigned a weight *ω* (*top*). Weights are integrated over the song (*thick black line*, without noise), and noise with standard deviation *σ* is added at each step to yield a noisy evidence (*grey lines*, five independent noise instantiations). A decision is fixed and integration stops after either the upper or the lower threshold (*θ*+ and *θ−*, respectively) is crossed (*red triangles*). If no threshold has been crossed by the song’s end, the response is given by the sign of the integrated evidence (*red* and *green circles*). **c** Model (predicted female response rate) vs behavior (actual female response rate) for all stimulus sets (*color coded*, see legend). **d** Model vs behavior separated by stimulus set (the same *color code* as in a). *Diagonal lines* in **c** and **d** correspond to a perfect fit

We hence used the experimental paradigm established previously for studying the decision-making processes underlying mate choice to now test a more diverse set of ten different subunits—including those resembling heterospecific songs, those only slightly deviating from the “typical” conspecific one and those that are expected to be particularly attractive (Fig. 1a). First, we designed song models with subunits that mimicked song features of two other *Chorthippus* species (i.e., *C. mollis* and *C. dorsatus*), expecting them to be weighted strongly negatively by the females to avoid futile mating with heterospecifics. Second, we chose song models that contained subunits with features that commonly occur in *C. biguttulus* songs but are known to be less attractive than the block-like song model chosen before (von Helversen and von Helversen 1994, 1997; Balakrishnan et al. 2001). Stimuli with a very short or shallow pause between subunits as well as those with gaps interrupting the sound pulses are regularly produced by conspecifics or could be the result of degradation during signal transmission. We hypothesized that such songs will be rated less negatively than those of heterospecifics. Third, we included stimuli with onset accentuation (Fig. 1a), since in natural songs, the onset of the song subunits is accentuated (Elsner 1974) and females responded more frequently to song models with onset accentuation than to standard subunits without accentuation (Balakrishnan et al. 2001). Remarkably, the height of onset accentuation found in natural *C. biguttulus* songs correlates with the size and immunocompetence of the males (Stange and Ronacher 2012; Ronacher and Stange 2013).

Applying the drift diffusion model to these data, we found that different subunit types are ranked according to the expected costs and benefits of mating: heterospecific song subunits exhibit the strongest negative weights, whereas syllables more closely resembling conspecific ones are weighted less strongly and include negative as well as positive weights. In addition, how information is integrated over the duration of an acoustic signal yields fast decisions that efficiently use sensory information.

## Methods

### Animals

We used virgin females of *C. biguttulus* L for behavioral tests. Animals were caught as larvae in the field or raised in the lab from collected eggs. After the adult molt, males and females were kept separately in large cages with ad libitum food (see Reichert and Ronacher 2015). Virgin females were tested between 1 and 2 weeks after the adult molt.

### Behavioral experiments

The test apparatus and testing procedure are described in detail elsewhere (Schmidt et al. 2008; Reichert and Ronacher 2015); here, only a short summary is given. *C. biguttulus* males try to attract females by producing calling songs that consist of 30–40 stereotyped noise “syllables” separated by short pauses (von Helversen 1972; von Helversen and von Helversen 1997). Important features for recognition and attractiveness reside in the species-specific amplitude modulation patterns of the songs (von Helversen and von Helversen 1997). Females respond to a male’s song if they are inclined to mate and if they judge the male song as attractive (Wirmer et al. 2010). This response behavior of females allows for an automated test procedure in which song models are played back to the female by a computer program (courtesy of Matthias Hennig) that also records the female response songs via a microphone (see Schmidt et al. 2008). The probability of registering a female response song estimated from many presentations of the same song (see below) is then taken as a measure of song attractiveness. By this experimental design, this study focuses exclusively on acoustic courtship signals, for other potential signals involved in mating decisions see “Discussion”.

### Stimuli

Each song model consisted of a mixture of two different subunits (see Clemens et al. 2014). A song comprised 33 subunits, corresponding to song durations of 2.5 to ~3 s, depending on the stimulus type (only for the “mollis” model, the total duration was longer, up to 7.9 s, due to the long syllables and pauses). Song durations above 2 s are well accepted by *C. biguttulus* females (von Helversen 1972, and own unpublished results). In a song model series, we varied the proportion of unattractive subunits from 0 to 100% in various parts of the song (the same mixtures as in Clemens et al. (2014)). A playback cycle consisted of 35 test song models that were presented in randomized order. Note that a female heard only one stimulus at a time—hence, she was subject to a no-choice paradigm. A playback cycle was always preceded by an attractive song model to guarantee female motivation. The playback cycle was repeated 18 times and the order of song models was newly randomized for each cycle presentation to minimize any potential carry over effects between stimuli. The fraction of responded presentations for each model song was taken as measure for its attractiveness. A 3-s unmodulated noise stimulus served as a negative control: females that responded more than two times out of 18 stimulus presentations to this negative control were excluded from further evaluation as unselective. Mean values of the response percentages from *N* = 12 to 22 females were evaluated per test series and served as the basis for the model fitting.

As standard stimulus of medium attractiveness, we used a song subunit consisting of a 72-ms noise “syllable” and a 12-ms pause as used by von Helversen (1972) and von Helversen and von Helversen (1997) for tests with *C. biguttulus* females. This standard song subunit is termed “block stimulus” (see Fig. 1a). Unless otherwise mentioned, the syllable plateau was presented at 64 dB SPL, while the pauses and gaps were silent. The block stimuli were combined with other stimulus types, as indicated below. In total, we tested ten different subunits (see Fig. 1a; Table 1 for details of the song parameters).

**Table 1:** Parameters of the song models

	Duration (ms)		Onset	Sound level (dB)		Onset
	Syllable	Pause		Syllable	Pause	
Negative stimuli						
Dorsatus	(8 + 6) × 4	40	–	8 = 64 6 = 0	0	–
Mollis	120	120	–	64	0	–
Offset (8 dB)	72	12	–	64	56	–
Pause (4 ms)	72	4	–	64	0	–
Onset (3 dB)	72	4	10	64	0	67
Offset accent	72	12	62–72	70	58	82
Positive stimuli						
Dorsatus	76	14	–	64	0	–
Mollis	72	12	–	64	0	–
Offset (8 dB)	72	12	–	64	0	–
Pause (4 ms)	72	12	–	64	0	–
Onset (3 dB)	72	12	10	64	0	73
Offset accent	72	12	10	70	58	82

- *Dorsatus vs block* The negative subunit consisted of four 8-ms noise pulses separated by three 6-ms gaps, and followed by a 40-ms pause, mimicking features of *C. dorsatus* songs (see Stumpner and von Helversen 1992). In this case, the standard block subunit was a 76-ms syllable followed by a 14-ms pause, to equalize subunit durations.
- *Mollis vs block* The negative subunit was a 120-ms syllable followed by a 120-ms silent pause. Such models are well accepted by *C. mollis* but not by *C. biguttulus* females (von Helversen and von Helversen 1975; M. Hennig, personal communication). The other subunit was the standard 72-ms syllable combined with a 12-ms pause.
- *Gaps vs block* We included data from a previous study (Clemens et al. 2014) with gaps in the subunit plateau that are generally rejected by females (Ronacher and Stumpner 1988; Kriegbaum 1989).
- *Offset 8* *dB (shallow pause) vs block* One subunit was a 72-ms syllable followed by a 12-ms pause at 56 dB SPL. Offsets of 8–9 dB are sufficient to detect a pause (von Helversen 1979), but shallow pauses are on average less attractive. Note that *C. biguttulus* males do produce noisy pauses in their songs (Elsner 1974; Balakrishnan et al. 2001). The other subunit as in (b).
- *Short pause vs block* A syllable of 72 ms was followed by a short pause of 4 ms; such stimuli are hardly attractive for females (von Helversen and von Helversen 1997). The other subunit as in (b).
- *Short pause with small syllable onset vs long pause with large syllable onset* One subunit consisted of a 4-ms silent pause followed by a 72-ms syllable with a small 3-dB onset accentuation (10 ms). The other subunit was a 72-ms syllable with an accentuated onset (9 dB for 10 ms), combined with a 12-ms pause.
- *Accent at the end vs accent at the beginning of syllables C. biguttulus* females prefer song syllables with an accentuated onset but dislike syllables that exhibit an accent at the syllable end (von Helversen and von Helversen 1998). As basis for the subunits, we used a 72-ms syllable with a 70-dB plateau followed by a 12-ms pause with 12-dB offset. One subunit had a (10-ms long) 12-dB onset accentuation; the other subunit was the time-reversed version with an accent placed at the end of the syllable (82 dB from 62 to 72 ms).

#### Structure of the drift diffusion model

To infer how each subunit was evaluated by the females and how this evaluation affected decision dynamics, we fitted a drift diffusion model to our data to predict the female response probability for each song stimulus. In a drift diffusion model, pieces of sensory information—in our case each subunit in a song—are assigned a weight and integrated noisily over the course of the song. The sensory weights can be either positive or negative, favoring or inhibiting a female response, respectively. The effect is exemplified in Fig. 1b for a song starting with 11 negative subunits followed by 22 positive subunits (arbitrary weights *ω−* = −2, *ω*+ = +1). The thick black curve in Fig. 1b corresponds to integration without noise. The grey curves are the result of five different runs with decision noise—at each time step, decision noise with standard deviation *σ* from a Gaussian distribution was added. The decision to respond or not is fixed either if a negative or a positive threshold is crossed (red triangles in Fig. 1b) or is given by the sign of the integrated information at the end of the song, in case no threshold was crossed (green and red circles at position 33 in Fig. 1b). The memory of the integrator is long compared to song duration in this species when tested with our stimulus paradigm, and sensory information, therefore, accumulates perfectly (see Clemens et al. 2014).

#### Model parameters and fitting

The parameters of the integrator—the noise level *σ* and the upper and lower thresholds (*θ−* and θ+)—were determined for all stimulus sets under the assumption that the integrator is stable across stimulus sets (Fig. 1c). This assumption was confirmed by the fact that allowing each stimulus pair to have its own integrator parameters did not increase model performance (supplementary figure S1). Possible stimulus-specific differences in the response behavior were reflected by differences in sensory weights. The weight of the most frequently tested standard block subunit was arbitrarily fixed to 1.0; the remaining nine weights were fitted to the data. The model parameters were fitted using a Genetic Algorithm as in Clemens et al. (2014). Model performance was evaluated using leave-one-out cross-validation. That is, model parameters were obtained using a training set of *N*-1 stimuli and the behavioral responses was then predicted for the held-out test stimulus. This was repeated until all *N* stimuli were in the test set once. All parameters were highly reproducible across different cross-validation runs—as indicated by the relatively small spread of parameter values in Fig. 1d—and generally well constrained by the data (Table 2 and supplementary figure S1). The threshold for negative decisions *θ−* was the only parameter constrained only up to a minimal value. This was due to the decision dynamics resulting from the relatively weak positive weights which required *θ−* to be only large enough to avoid too early fixing of negative decisions but did not set a strict upper bound on its value.

**Table 2:** Subunit weights and integrator parameters

Dorsatus-like	Mollis-like	Gap	Offset (8 dB)	Pause (4 ms)	Onset (3 dB)	Onset (9 dB)	Accent offset	Accent onset
−82 ± 6	−87 ± 6	−55 ± 4	−51 ± 4	−53 ± 5	−40 ± 3	15 ± 2	−34 ± 2	7 ± 1

Noise	Upper threshold	Lower threshold
142 ± 6	420 ± 26	−803 ± 81

See “Methods” and Fig. 1a for a detailed description of each subunit. Values correspond to mean ± standard deviation over all 218 cross-validation runs. Note that the onset 3 dB syllable was combined with a 4-ms pause that rendered this stimulus unattractive (see “Methods”)

## Results

### A drift diffusion model reveals differential evaluation of song information

The drift diffusion model reproduces the responses very well for each individual data set (*r* ^2^ between behavior and model over the full data set is 0.87, Fig. 1c, d). The model parameters were well constrained by the data and reproducible across different runs of the model fitting procedure (Table 2, supplementary figure S1).

**Fig. 2:**
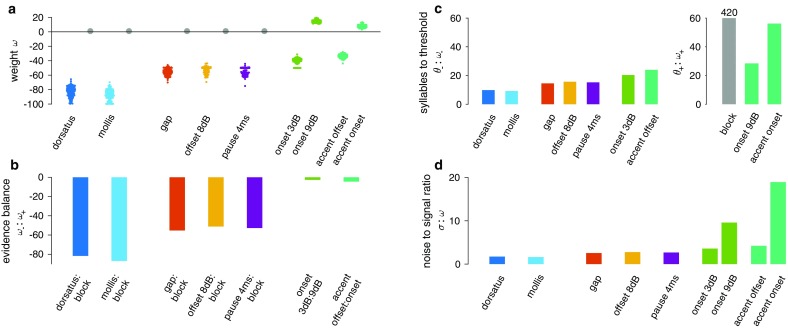
Subunits weights. **a** Weights for each subunit in our data set, sorted by the stimulus set they were presented in. Each *small dot* corresponds to the estimate obtained from each cross-validation run (*n* = 218). *Big grey dots* correspond to the block subunit with weight fixed to 1.0. The two heterospecific subunits (dorsatus and mollis) have the most negative weights, while subunits sharing features with conspecific songs are weighted less negatively or even positively. **b** Balance between positive and negative evidence, given by the ratio of the weight for the negative and positive subunits (*ω*−/*ω*+) of each stimulus set. All values are smaller than −1.0, indicating that the negative subunits always outweigh the positive subunits. **c** Number of successive subunits in a song necessary to cross a threshold, given by the absolute ratio of each weight to its threshold *θ*: |*ω−*/*θ−*| (*left*) or |*ω*+/*θ*+| (*right*), respectively—for all pairs of subunits tested. The value for the block subunit in the right panel (*grey*) was 420 and the *Y*-axis was cut to highlight differences between the remaining subunits. Note that the onset-3 dB syllable was combined with an unattractive 4-ms pause (see “Methods”). In general, subunits with negative weights (*left*) reach threshold after integrating fewer subunits than the positive subunits. **d** Noise-to-signal ratio, given by the ratio of the integrator’s noise *σ* and each subunit’s weight *ω*. All values are >1.0, indicating noisy integration. However, the positive subunits (onset 9 dB and accent onset) have especially high relative integration noise

We first examined the weights *ω* associated with each subunit, since they directly reflect the value females assign to each subunit (Fig. 2a). Consistent with our prediction, the two model subunits resembling songs produced by a different species—*C. dorsatus* and *C. mollis*—are assigned strongly negative weights (−80 to −90). By contrast, conspecific cues are weighted in a graded manner: Five subunits associated with the conspecific range of song parameters—gap, offset 8 dB, pause 4 ms, onset 3 dB (with 4 ms pause), accent offset—have smaller negative weights (−60 to −30). Two of the subunits tested here—onset 9 dB and accent onset—are associated with moderately positive weights (5–15) when compared to the standard subunit whose weight was fixed at 1.

### Negative evidence dominates the decision process

Although there exist more positively weighted subunits, negative evidence is dominant in driving decisions (Fig. 2b). In the stimulus sets with the two heterospecific subunits, negative information clearly dominates the decision (*ω*−/*ω*+ ≈ −80). However, even for the stimulus sets with the most positively weighted subunits—onset 9 dB and accent onset—negative evidence still outweighs positive evidence (*ω−*/*ω*+ = −2.7 and −4.5, respectively). The dominance of negative evidence is also apparent when comparing the positive and negative sensory weights to their respective thresholds (Fig. 2c). The negative threshold is at ~−800, and hence, only ten heterospecific subunits suffice to fix a negative decision (without noise). By contrast, 29 of the most positive subunits (onset 9 dB) are required to fix a positive response by threshold crossing. In all other cases, positive evidence alone is unable to reach the upper threshold before the song ends.

Integration in our model is noisy and the signal-to-noise ratio—the quotient of the sensory weights *ω* and the noise level *σ* (Fig. 2d)—determines whether decisions are mainly driven by noise or by sensory information. The noise level in our model is ~140 and hence 1.7–4.2 times stronger than negative evidence and 10–19 times stronger than the positive evidence. For the standard stimulus without onset, the noise exceeds *ω*+ by a factor 1:140. Hence, as shown previously, noise strongly influences decisions for all subunit types—especially the integration of positive evidence (cf. Clemens et al. 2014).

### Integration dynamics

**Fig. 3:**
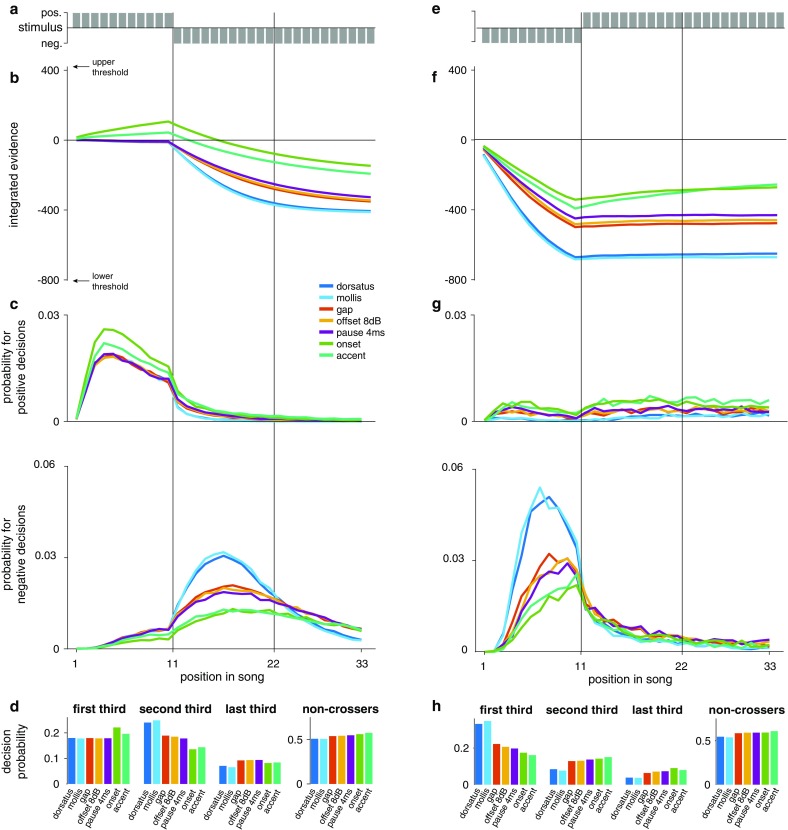
Integration dynamics. **a**, **e** Example songs in which the subunit type switches after one-third (11 subunits) from positive to negative valence (**a**) or vice versa (**e**). Each syllable is indicated by a *grey bar*. **b**, **f** Integrated evidence of the song in **a** and **e** for all pairs of subunits tested (*color coded*, see legend in **c**). For plotting purposes, we averaged the evidence over 50,000 repetitions, since integration is noisy. *Y*-axis limits correspond to the upper threshold and lower threshold (420 and 803, respectively). **c**, **g** Probability of fixing positive or negative thresholds (upper and lower parts, respectively) during the song for each pair of subunits tested (see legend for *color code*). Differences in the decision dynamics are most prominent before (**g**) or after (**c**) the subunit valence switches in the second third of the song (**a**, **e**). **d**, **h** Probability of crossing the threshold during the first, second, and last third of the song (first three panels) and of not crossing threshold by the end of the song (last panel) for each subunit (the same *color code* as in **c**). If no threshold is crossed, the decision is given by the sign of the integrated evidence at song end

Given the poor signal-to-noise ratio of evidence integration, we asked to what extent the observed sensory weights affect actual decision dynamics—for instance when a decision is fixed during a song. To that end, we examined the time course of integrated evidence and decision times for an example song in which the valence of subunits switches from positive to negative after the first third (Fig. 3a–d). The integrated evidence (average over 50,000 repetitions) reflects the differences in weight between stimulus sets (compare Fig. 3b): for the two subunit types with onsets, the positive evidence increases considerably during the first third, while the remaining stimulus sets with the neutral standard subunit hover around zero. Thus, the two stimulus sets with positive evidence start with a clear positive bias when negative subunits occur later in the song. Integrated evidence then decreases according to the weight of negative evidence for each song type, with the heterospecific subunits reducing the integrated evidence most strongly. The integrated evidence does saturate (see, e.g., light blue line) due to the thresholds—after threshold crossing, values are fixed at that of the threshold.

Surprisingly, the differences in the integrated evidence only incompletely translate into differences in the decision dynamics (Fig. 3c). The probabilities for a positive decision—resulting from decision noise—are in the range of 2%. Note that in the first third, there is also a non-zero probability for negative decisions, i.e., crossing of the negative threshold, despite only positive subunits being present (Fig. 3c, lower graph). Figure 3d represents the overall decision probabilities integrated for the three sections of the song. Remarkably, the two most positive subunits–onset 9 dB and accent onset—yield only a marginal increase of threshold crossings within the first third compared to the other stimuli (Fig. 3d, first third, green and turquoise bars), because the positive evidence is too weak to drive the integrated evidence to threshold (Fig. 3c, compare Fig. 2c). However, negative decisions are clearly reduced for these two stimulus types after the switch to negative syllables (Fig. 3d, second third). This indicates that the change in the balance between positive and negative evidence (Fig. 2b) is not sufficient to strongly drive a positive decision; rather, it delays negative decisions by preventing early threshold crossings, leading to a more complete evaluation of the whole song. By contrast, the dorsatus and mollis stimuli exhibit increased decision probabilities in the second third, reflecting the increase of negative threshold crossings. A rather large proportion of decisions (around 50%) in this example is not due to a threshold crossing but depends on the sign of the decision variable at the end of the song (“non-crossers” in Fig. 3d). In Fig. 3e–h, responses to the complementary stimulus—starting with 11 negative subunits, followed by 22 positive ones—are investigated. Here, a strong vetoing impact of the negative subunits is evident for all subunit types (Fig. 3f), while after the switch to positive subunits, the integrated evidence remains almost constant. Figure 3h represents the integrated decision probabilities for the three sections of the song. For the two heterospecific subunits, mollis and dorsatus, the integral probability to reach the lower threshold during the first third is particularly high, ~0.35.

## Discussion

Sexual selection theory predicts that courtship signals should be evaluated according to the costs and benefits associated with accepting the sender as a mate. Here, we tested this hypothesis in the context of song evaluation by female grasshoppers. A model-based analysis of behavioral responses to playback of ambiguous song information (Fig. 1) revealed how different song patterns are evaluated by the female (Fig. 2) and how this evaluation affects decision dynamics (Fig. 3) and ultimately mate choice.

The weighting of the different stimulus types was consistent with our hypothesis. Stimuli that mimicked the songs of a different species (*C. mollis* and *C. dorsatus*) were valued most negatively (Fig. 2a). These very strong negative weights lead to an early and robust rejection of heterospecific males (Fig. 3d, h), mating with which would compromise female fitness. Stimulus features frequently occurring in the songs of *C. biguttulus* but less strongly deviating from the species-typical pattern (von Helversen and von Helversen 1997) are weighted less negatively than the heterospecific ones (Fig. 2a). However, they still lead to a clear rejection (Fig. 2c), probably because these deviant song features—gaps and very short or shallow pauses—indicate poor male quality (Kriegbaum 1989; von Helversen and von Helversen 1994). For instance, gaps in the songs are produced if a hind leg is lost or a forewing is crippled due to difficulties during the molt—loss of a hind leg may also occur as a result of predation (Elsner 1974; Kriegbaum 1989). The occurrence of gaps can also result from loss of the left–right coordination or from a malfunction of the central pattern generator that controls the stridulation movements (Elsner 1975; Hedwig 1992). Similar deficits of the central pattern generator may produce songs with a reduced pause depth or songs with very short pauses (Ronacher 1989). Females are known to reject such stimuli (Kriegbaum 1989; von Helversen 1979), thereby they avoid mates with developmental problems indicative of inferior genetic quality (Qvarnström and Price 2001; Ritchie 2007).

The two subunits with the most positive weights (Fig. 2a) exhibited an onset accentuation (Fig. 1a). Interestingly, natural songs of *C. biguttulus* males normally do exhibit an onset accentuation of the syllables in the range of 5–9 dB (Elsner 1974), the height of which correlates positively with male size and immunocompetence, suggesting that onset accentuation is an honest signal of male condition (Stange and Ronacher 2012; Ronacher and Stange 2013). The higher weight of these subunits (Fig. 2a) thus may reflect a selection for high-quality males.

However, even for the most attractive subunits, sensory information only weakly biased the integrated song information and was barely able to drive positive decisions by threshold crossing (Fig. 2c). The main function of attractive subunits in the model is to prevent the early fixing of negative decisions and to allow integration to carry on until song end, so that the sign of the integrated evidence can trigger a response (Fig. 3d, h). The overall bias against the decision to mate—as reflected in the small weights of attractive subunits (Fig. 2a)—may reflect baseline costs of mating for the female (Head et al. 2005; Kotiaho and Puurtinen 2007) or could be associated with the fact that eggs are a limited resource for the female (Kriegbaum 1997). An additional or even alternative reason for the surprisingly low effectiveness of attractive stimuli may lie in the restriction of our test paradigm to the acoustic signal domain. For several grasshopper species, visual signals are crucial elements of the courtship (e.g., Elsner and Wasser 1995), and recently, a strong impact of chemical cues on mating decisions of *C. biguttulus* has been demonstrated (Finck et al. 2016a, b; Finck and Ronacher 2017). Thus, additional multimodal stimulation may change the balance between positive and negative weights.

Preferences for mating signals may depend on the signals experienced before, as has been demonstrated in crickets (Bailey and Zuk 2009). Hence, we must consider how the different sets of tested stimuli may have influenced the response propensities. First, we can exclude an influence from one stimulus set to another, since each set was tested with a separate cohort of females. We included the identical block stimulus in most sets to have a standard for comparisons, and in the model fitting, the weights of all other stimuli were related to this standard that was given the weight *ω*+ = +1. Within a stimulus set, the influence of one stimulus to the next cannot be eliminated but was minimized by newly randomizing stimulus order for every presentation cycle (see “Methods”).

The overall dominance of negative information in the decision process (Fig. 2b) and the great utility of rejecting songs with non-attractive features raise the question whether negative information is simply a lack of positive cues or whether there exist dedicated detectors in the female’s nervous system for unattractive song properties. Consistent with the latter idea, a modelling study on feature detectors has revealed a unit with a suppressive effect on female responses (Clemens and Ronacher 2013; Ronacher et al. 2014). Moreover, a neuron with response properties ideally suited for gap detection has been described earlier (Ronacher and Stumpner 1988; Franz and Ronacher 2002), suggesting that there exist dedicated circuits for detecting negative cues.

Our model-based analysis reveals that females do indeed evaluate different subunits according to the expected costs and benefits of mating. The ensuing decision dynamics appear to be tuned for fast and accurate decisions: If there is a cost to waiting, early commitment to a decision in the case of clear evidence is efficient (Tajima et al. 2016; Thura et al. 2012; Drugowitsch et al. 2012). However, if the evidence is less clear, all available information should be considered to improve decision accuracy. In our case, negative sensory evidence has a strong vetoing effect—a few negative subunits suffice to drive the decision variable to a rapid rejection of the song (Fig. 3c, d, see also Clemens et al. 2014). In contrast, positive evidence keeps the integration process going by preventing an early commitment to a rejection (Fig. 3c). This will also prevent the female from responding to heterospecifics that sing right after a conspecific, since a single unattractive syllable at song end can drive the integrated information to negative values and veto a response. The dynamics of decision-making are thus tuned to speed up decision-making for unambiguous information and to utilize all available information completely when a more graded assessment of the male is required. In the light of efficient decision-making strategies, it is unclear why the integration process is so noisy (Fig. 2d). Such high noise levels may reflect physiological constraints—e.g., low firing rates in the neurons integrating information at higher processing stages. Indeed, sparse coding has been reported for crickets and grasshoppers (Kostarakos and Hedwig 2012; Clemens et al. 2011, 2012).

Overall, our results support the hypothesis that the rules driving mate choice decisions reflect selective forces, in particular the strong incentive to avoid crossbreeding with another species which would lead to severe losses in reproductive success (Gottsberger and Mayer 2007; Finck and Ronacher 2017). It will be interesting to apply our framework for testing this hypothesis in other mating systems that exert different costs to mating. For example, females which receive direct benefits from mating in the form of nuptial gifts (Lehmann 2012) may exhibit a positive bias to mating and the decision process may thus unfold differently.

## Electronic supplementary material

Below is the link to the electronic supplementary material.

Supplementary material 1 (DOCX 204 kb)
