# Supplementary material for: Relative weighting of acoustic information during mating decisions in grasshoppers indicates signatures of sexual selection
Source: J Comp Physiol A Neuroethol Sens Neural Behav Physiol. 2017 Jul 21;203(11):891–901. doi: 10.1007/s00359-017-1200-x (PMC5644699; doi:10.1007/s00359-017-1200-x)
Supplement: Supplementary file 1 — Supplementary material 1 (DOCX 204 kb) [file 359_2017_1200_MOESM1_ESM.docx]

**Supplemental Figure 1**

**a** All parameters are well-determined by the data. Shown is fold change of the mean squared error for fold changes in each parameter value on a log2 axis. Grey lines correspond to the MSE for 16 independent realizations of the noise.

**b** A model with integrator parameters (noise, upper and lower threshold) individually fitted for each pair of subunits tested (x-axis) does not outperform the model with one set of integrator parameters for all subunits (y-axis) (p=0.45, sign test). Data points correspond to the different pairs of subunits tested (see figure 1a).
